# Problem-solving for STEM learning: navigating games as narrativized problem spaces for 21^st^ century competencies

**DOI:** 10.1186/s41039-016-0038-0

**Published:** 2016-07-07

**Authors:** Azilawati Jamaludin, David Hung

**Affiliations:** National Institute of Education, Nanyang Technological University, 1 Nanyang Walk, Singapore, 637616 Singapore

**Keywords:** Game Play, Affinity Space, Discursive Practice, Structural Dialectic, Game Space

## Abstract

Identifying educational competencies for the 21^st^ workplace is driven by the need to mitigate disparities between classroom learning and the requirements of workplace environments. Multiple descriptors of desired 21^st^ century skill sets have been identified through various wide-scale studies (e.g., International Commission on Education for the 21st Century) and consistently within the context of science, technology, engineering, and mathematics (STEM) learning, the ability to problem solve, particularly complex problem-solving, remains a crucial competency. In this paper, we look at how current contemporary spaces such as the immensely popular, massively multiplayer online role-playing game(MMORPG), World of Warcraft, (WoW) afford problem-solving skill acquisition in the context of Singaporean youth learners. Given that WoW exists as a contextual space with an overarching narrativized problem to be solved, our investigation focused on two important related constructs that underpin learners’ problem-solving trajectory—learning and identity *becoming* within contemporary domains of technology learning. We present findings of an ethnographic investigation of one youth gamer within the affinity spaces of WoW. Moving away from traditional mentalistic construals of problem-solving, our findings indicate that problem-solving within WoW may be characterized by a triadic-D model of domain, disquisitional, and discursive practices within self, social, and structural dialectics. Theoretical considerations for broadening the understanding of a situated and embodied notion of problem-solving and identity *becoming* within STEM learning are proposed.

## Complex problems of the 21^st^ century

The twenty-first century learning evolves from new cultural forms of digital literacy and marks a significant shift from the conventional accessing of information to solving routine problems. Rather, contemporary work environments revolve around the management of complex information streams aligned with complex problem-solving tasks that require expertise across multiple cultures of science, technology, engineering, and mathematics (STEM) learning. Complex problems may be characterized as typically ill-structured, with unknown elements or elements not known with any degree of confidence (Reitman 1964; Wood 1983). Many interrelated factors dynamically affect the problem states creating different representations and understandings of the problem (Funke 1991). In facing a complex problem, mere possession of theoretical knowledge but without its attendant application experience poses a problem in itself. This is so as “real-world” problems are inherently ill-structured and seldom have a single, best solution; they typically possess multiple solutions or no solution at all (Kitchner 1983). Wherein institutionalized learning has been very successful in equipping students with producing the “correct model” solution, the de-contextualized nature of schooling seeds incongruence between content and culture—students learn content that is framed within the culture of schooling, but whose application is oriented towards a target professional domain and its attendant complex problem paths. Such incongruence is further compounded by the current “complex” landscape of interaction in which the youth engage the world and each other.

Within contemporary narrativized problem spaces such as World of Warcraft (WoW), youths are developing sophisticated competencies coupled with new cultural norms and social practices inherently disparate from that of school practices (Jamaludin, Kim, and Hung 2012). As they go about engaging in solving the tasks and quests afforded by the WoW game space, the multiplayer aspect renders that players’ performance within the activities transacted are non-solitary. Rather, the nature of interaction within the game space is underpinned by an intertwining relationship between individual performances and the interaction and collective emergence of social communities. For example, just as problems within real-life or workforce settings are complex in such a way that they may be solved when people of differing expertise and specializations converge to contribute and develop a functional solution, so too are the “problems” within WoW—players operate at a collaborative level with varying expertise to achieve the gameplay objectives (e.g., end game raids). Not only do they have to understand their own role within their chosen class (e.g., the strengths and weaknesses of a Paladin), but their game play advancements also hinge on their ability to synergize with players of other classes (e.g., hunter, warrior, shaman) This calls for not only an intimate understanding of the player’s own strengths and weaknesses but also a cognizance of oneself in relation to the social *other*. The success or failure of such self-socio-interplays is rapidly evidenced within the game through twofold trajectories of *questing* and *raiding* where success is represented by uplevels and failure by group wipeouts. While questing involves semi-defined problem quests embedded within the gaming narrative that each player has to “solve” to level up or move to other more advanced areas of the game, raiding involves a competitive interaction among players requiring large group of players to come together and collaborate in defeating computer-generated monsters. Problems embedded for the latter are more complex in that greater sophistication of player strategies are required to address dynamically interrelated factors such as the strength of a raid group (determined by the appropriate number of tanks, healers, and damage dealers class characters) and knowledge of “boss” fights (determined by a synergization of players’ experience points).

In a sense, a higher degree of sociality in terms of communication, collaboration, and cognizance of oneself as *another* is needed to push forth optimal raiding solutions as a group. These cycles of individual performances and co-evolved interactions as players make meaning and self-regulate learning in the context of technology-facilitated learning represent the central focus of our research. Through investigating how players, as learners, structure their cognitive development, construct and negotiate their identity and sense of self, and make meaning while navigating a fundamentally “problem-based” context, we aim to illuminate the problem-solving processes emerging in such technology-facilitated environments. To this end, our investigation focused on two important constructs that underpin learners’ problem-solving trajectory (Anderson 1980; Jonnassen 2000)—identity *becoming* and cognitive development in terms of learning and appropriation. As part of a larger study on investigating identity becoming trajectories of youth gamers (Jamaludin and Hung 2014), this paper presents our ethnographic investigation of one youth gamer across the affinitive problem-based spaces of WoW. An analysis of the problem space shows the dialectical relations between self, social, and the structural dialectics that are largely shaped by domain, disquisitional, and discursive practices. Theoretical considerations for broadening the understanding of identity and problem-solving are proposed.

## Problem-solving and identity *becoming* in problem-based MMORPGs

Contemporary youth culture such as active engagement in massively multiplayer online role-playing game (MMORPG) activities reflects the growing collection of technologies within popular culture that is profoundly affecting the development and expression of human lives (Levinson 1998) and broadly engaging the multiple “I” that are in dialogue with other selves and the cultural world. Within the context of youth’s online phenomena, the socio-spatial dimensions that underpin the development of youth’s meaning-making experiences create interrelated dynamics that not only afford youth the agency for explorative emplacements (Soja 1993) but also bear upon their identity trajectories. For example, in the MMORPG of WoW, players may reenact the social expectations of particular identity figurations or invert those expectations through specific character choice—they may select different modes of gender identification; therefore, it becomes possible to subvert expected gender roles. In a sense, the power of the game such as WoW lies not in the singular narrative of the developer but in the multiple narratives constructed by the player as he or she goes along, reflecting the ongoing performance of one’s complex and flexible self. In this conception, one’s self is constituted of contradictory identities pulling in different directions, so that identifications are continually being shifted (Barker 2008). At the same time, the player’s performance of self within such structures also hinges on identifying with an individual customized character—either through varied subject positioning, identification with the given character in a number of different ways, or character customization followed by role-play identification (Hutchinson 2007). In such configurations, a sense of agency comes largely from freedom of movement (Carr 2003) both spatially and socially (Turkle 1995; McBirney 2004). It is not surprising then that the youth living in postmodern conditions are inevitably drawn to the online worlds of WoW, as the appeal of these spaces pivots around the agency of choice, strongly coupled with the socially oriented tradition of MMORPGs (Jamaludin and Chee 2011).

Closely related to these shifts is the intertwining issue of learners’ identity development referred to as *becoming*. Identity in this sense plays out in terms of self-understanding as bound to particular contexts, practices, or environments. Traversing between digital spaces and the schooling milieu, identity from a social constructivist point of view is not definitive in that an individual possesses only one identity, but rather identity is multiple (and situative) and dialogically constructed. The phenomena in MMORPGs are consistent to situated cognition where human knowledge and interaction cannot be divorced from the world (Lave and Wenger 1991). As observed by Thomas and Brown (2011), the bounded context of WoW represents the affinity space for knowledge and technology-facilitated interactions to be co-determined. The social construction of self-knowledge mediated by identity *becoming* thus cannot be learned without coupling to the contextual community. Viewed as an experience of identity, learning in this sense entails both a process and a place. It entails a process of transforming knowledge as well as a context which defines an identity of *participation*.

Implicit to this relationality, we recognize that players—as learners—are perpetually in a creative state of *becoming* as they traverse across multiple contexts of interaction. *Becoming*, in Deleuze and Guattari’s (1987) sense, is a non-teleological, continuous process through which any entity may make rhizomatic connections to other things that matter. A *becoming* is “neither one nor two, nor the relation of the two; it is the in-between” (Deleuze & Guattari 1987, p. 293). Within an experiential, problem-based space such as WoW, as members engage in the *becoming* trajectories, they are operating at both levels of personal (individual play) as well as collective (guild or raid instance) enactments. These levels, however, are not merely an aggregation, in that the social effects at the collective level cannot merely be described as individual contributions and volitions at the personal level. Rather, an interaction at these two levels may more aptly be characterized as dialectical and an emergence of rhizomatic relations embedded in and performed by shifting connections and interactions (Jamaludin et al. 2012). Such relations are rhizomatic in the sense that O’Riley (2005) describes them as “dynamic, heterogeneous, and nondichotomous; … they propagate, displace, join, circle back, fold … de- and reterritorialize space” (p. 27) where there is a non-hierarchy of multiple narratives, without origin or central root to serve as a source. Pragmatized by Deleuze and Guattari (1987), rhizomatic relations thus foreground and value connections over nodes and always begin in the middle and problem-laden muddle of life, a situation that is prevalent among our youths in their current rapidly and changing contexts. As Lave and Wenger (1991) summarized, rooted within a social context, “learning thus implies *becoming* a different person with respect to the possibilities enabled by these systems of relations. To ignore this aspect of learning is to overlook the fact that learning involves the construction of identities.” (p. 53). In this regard, gameplay in MMORPGs in WoW may thus be characterized as processes of *becoming* against a system of relations, arising from a narrativized problem context.

## Disrupting traditional construal of problem-solving in MMORPGs

Theories of problem-solving have traditionally placed focus on individual characteristics such as self-efficacy, goal setting, and achievement. For instance, cognitive psychologist Wallas (1926) developed a four-stage model of problem-solving explicated as (1) preparation—defining the problem and gathering information relevant to it; (2) incubation—thinking about the problem at a subconscious level; (3) inspiration—having a sudden insight into the solution of the problem; and (4) verification—checking to be certain that the solution was correct. Similarly, Polya (1954) described the process of problem-solving in the following four steps: (1) understand the problem, (2) devise a plan, (3) carry out the plan, and (4) look backward. Newell and Simon (1972) proposed a theory of human problem-solving that emphasized the similarities between artificial intelligence and human problem-solving. They articulated four underlying principles of this theory: (1) a few gross characteristics of the problem-solving process are invariant over the task and the problem solver, (2) the characteristics of the problem are sufficient to determine the problem space, (3) the structure of the task environment determines the possible structure of the problem space, and (4) the structure of the problem space determines the possible programs (methods) that can be used for problem-solving. While we recognize the salience of the articulated steps and structures in problem-solving processes, we argue that at an underlying level, such theories remain mentalistic and individualistic—much emphasis remains on the recognition of one’s own epistemic orientations and habits of mind, with a disregard for one’s embodied self in relation to others. This stands in contrast to the kinds of problem-solving activities observed in WoW.

**Fig. 1:**
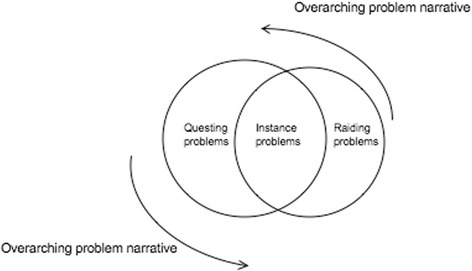
Problem structures in a WoW narrativized space

In the WoW gaming space, a narrative structure exists to explicate the overarching *problem* players are faced with—the hordes and alliance races are in feud and there is a need to restore peace in the land of Azeroth. In attempting to achieve the utopian stage where races are at peace, players are expected to move through trajectories of game play where they are faced with: (i) problem quests at different levels of the games, (ii) instances problems where they have to come together in groups of between five and ten players, and (iii) raiding problems in groups of 25 or more players before they are able to reach the end-game stage. Figure 1 illustrates the narrativized problem structure of WoW.

In a sense, through embodied avatarial interfaces, players are frequently engaged in non-linear functions of learning that are inherently problem-based. For example, in undertaking a specific quest, players are presented with a problem to be solved where the process to achieve goal involves exploring (e.g., of delineated worlds in games), communicating (e.g., of specific game lingo), regulating (e.g., of a way of interacting with the social others), and strategizing (e.g., of rapid wipe techniques for the next raid). In taking on different characters through various embodied representations, one’s game play problem-solving experience may essentially be construed as a deeply embedded practice that is intimately linked to his/her identity construction processes rooted within a social context. Given the limited research that takes into account the recognition or know-how of cultural-contextual structures assumed in the social others and in the cultural resources/environment for processes of problem-solving, we argue that there is a need to address this gap through a shift towards a more structurally situated and embodied view of problem-solving.

This paper is therefore an expedition to unpack constructs underpinning players’ situated and embodied problem-solving trajectory, a key competency for STEM learning. Specifically, we draw upon one player’s experience to illustrate the identity *becoming* and learning processes as a player navigates the narrativized problem spaces of WoW. Learning in game play spaces such as WoW has been recognized to potentially arise from the interaction between both the game play (that is, the play structured by the game as software) and the social practices going on in and around the game. Gee and Hayes (2010) characterized the kinds of social structures underlying interests in the game as affinity spaces. For example, WoW players are engaged not only within the game but they also converge in fan sites or forum sites to further interact, discuss, and analyze WoW based on a shared affinity for the game. As highlighted by Gee and Hayes (2010), affinity spaces differ from communities of practice (CoP) in that the former characterizes fluidly populated social groupings that entail a continuum of membership from short-term lurkers to ardent game aficionados. While membership to CoPs entails a minimal participatory investment (e.g., at the legitimate peripheral level), participation boundaries in affinity spaces are less structured in that “membership” includes even a fan site visitor who has come only once to the site as being in the affinity space and therefore part of what defines the space. In tracing players’ problem-solving trajectories, we take a synchronic view that looks across the affinitive spaces that the WoW player “participates” in on a regular basis, which includes the player’s game play in WoW and the player’s WoW forum, guild site, and social media affinities (Facebook and YouTube).

## Method and data analysis

In this paper, we present the findings of our ethnographic study into the problem-solving activities of one youth gamer. Our investigation was focused on (1) the construction and negotiation of the player’s identities and (2) player’s *becoming* (in terms of identity and learning) trajectories as he engaged in the various problem-solving processes. At an initial level, identification of participatory spaces that the player frequently engaged in, in relation to WoW, were conducted. Based on Wenger’s (1998) work, these spaces were identified in terms of the specific discourse topics, for example, focusing on game strategy regarding the best approaches to in-game problems. Multiple data sources of interviews, field observation notes, instant messaging transcripts, and social media postings were employed to establish data triangulation. Face-to-face interviews with the focal player were conducted at four point intervals (i.e., time 1, time 2, time 3, and time 4) across a period of 1 year. These intervals corresponded to the first, second, third, and fourth term break (after 10 weeks) of the player’s school year. In addition, communication was sustained with the focal player through the use of technology-facilitated social media tools such as Facebook and Twitter. Field notes were used to record the focal student’s behaviors, actions, and interactions within the respective spaces. In-game chat logs were captured using a third-party game add-on called ChatLogger while enactment frames and videos were captured via Fraps. In terms of data analysis, a constant comparative method (Strauss and Corbin 1998) was employed to identify salient components and characteristics. In comparing gaming transcripts and interview data, our emerged analytical categories were coded as follows: domain practices, disquisitional practices, and discursive practices. Within the context of this study, domain practices, in Gee’s (2003) semiotic domain sense, were defined as those which bear upon the construction of domain identities, such as learned norms and the merging of goals and values that merged with the player’s own. Disquisitional practices referred to more structured reifications of the player’s game play processes while discursive practices were verbal descriptions used by both the player and his social others with regard to the player’s identity in terms of his performance and competencies. To achieve a balance of data presentation, the constructed identities (i.e., domain, disquisitional, and discursive identities) were voiced by the *self* as well as through the perspectives of the focal player’s social *other*.

The focal player in this study was 14-year-old Danny (pseudonym), a male student in secondary 2, equivalent to grade 10 of a local public school. His foray into online games started since the age of ten and he spends up to 6 h daily online. His first game was Maplestory, introduced by his best friend in primary school. Danny was selected because he was a relatively new WoW player which enabled us to trace his trajectory as he leveled up within the game space. Our initial observation and informal conversations with Danny uncovered somewhat related challenges that he faced. For example, being a relatively new WoW player, Danny had the ability to immediately understand the interactive properties that account for movement and positioning within the game environment; yet, he struggled while orienting himself to the game constraints and affordances needed to level up. In the following section, we further provide an analysis of Danny’s constructed and negotiated identities as he engaged in problem-solving based on the constant comparative categories of domain, disquisitional, and discursive practices.

## Problem-solving and (semiotic) domain practices

We observed that although the narrativized problem-based structure in WoW is characterized by tractable problems spaces within the respective questing zones, there is rarely one optimal trajectory to the solution. Instead, the meaning of problem-solving in the WoW context hinges on successfully solving a plethora of related ill-defined problems such as the synergization dynamics between the various damage and healer class, and honoring in-situ player honor codes while raiding—what may be termed as the semiotic “grammar” (Gee 2007) of gameplay. In one paladin-class quest instantiation, Danny was observed to have engaged in a quest that was currently being attended to by another player within the space. As non-player character objects were “slain” in the quest, Danny moved forth to collect the “loots” (winnings from a successful quest solved) arising from the mentioned quest. Although there are no explicit rules to prevent a player from doing so, Danny’s enactment (in claiming loots from a quest he did not initiate) is viewed as one that is a violation of the “norms” within the gameplay. Consequently, an antagonistic moment arose between Danny and the other quester wherein Danny was labeled a “noob” and an “impeder” (Text 1).

### Text 1

- Interlocutor: get out of my way you spastic noob. Those are my gold.
- Danny: there’s no law saying I can’t take
- Interlocutor: you are obviously an idiot. You want gold, you get your own bl**dy quest. don’t live off me. Your XP sucks and obviously you are zero DPS type impeder
- Danny transports away from the arena.

In a post interview with Danny (time 2), we surfaced this antagonistic exchange between him and his interlocutor, and Danny portrayed a nonchalant attitude towards gaming norms and practices.

### Text 2

“…that was one of my first quest, and when I saw gold, I immediately took them, but then that guy called me a noob and idiot. Quite aggressive, he kept poking me to duel so I went off. Then my friends told me I cannot be like that. My friends, they are already advanced and they like to tell me what to do and how to play but I was like heck... I continued”.

However, in a later post interview with Danny (time 4), the nonchalance towards gaming “norms” was observed to have shifted into a cognizance for what other players may construe of his game play strategies in solving the quests.

### Text 3

Danny: “…posted about me on the wowhead and some people remembered… Its like sometimes I go co-quest, then some of them tell me don’t take my loot and they guard fiercely then I can’t move and I ask them why bother. And last time I didn’t know what he’s talking about. Now I can program my own mount path”.

In tracing his gaming trajectory, we observed that beyond time 2 (where collaboration for questing and instantiation objectives intensifies), Danny evolved to manifest a strong affiliation with his character class. For example, he posted on the WoW forum space his explications and “debriefings” of the instantiation episodes he experienced and even theorized about the problem space, in terms of simulating specific “quest helpers” to speed up the “loot” attainment process.

### Text 4

Danny: …“gala, in getting the marines not to sink the voyager, you have to 50dps up one level while I remain below. You need to do your Math. We are not going to hit 4 k dps if your XP goes down ¾. Look at markam’s guide, I think we have two options here. 1) if you want the cross projectile to land just below, I think you have to aim behind 40, 55 (check your carto). Quite standard right? Gravity pulls you down. 2) go via kreekoak 22, 41 we can hit the front asypiate, or go behind take off the ramp, and you push off 157, 33. From qc99 you can get this”.

The shift in his stronger identification with his character and its strength and weaknesses is coupled with his increasing skills within the game, that is indicated by his leveling up paths (game level), his experience (XP) points, as well as the sophisticated armory and mount which he managed to obtain. At time 4, Danny has managed to achieve level 65 status, with a high level of 85 % experience points. In asking to him to explicate his view of himself as a WoW player, Danny cited not only his level and experience but also several instantiation accounts where he aided other players in solving the quests and attaining the sought-after loots. Apart from manifesting an awareness of being able to communicate within the genre of WoW, utilizing acronyms and game-specific terms, we observed that Danny is also able to acknowledge and align himself with the cultural norms of the gaming space and in sharing seemingly consistent values of achieving end-game raids through non-violation of player’s (tacit) honor code.

## Problem-solving and discursive practices

The game design affordances and the narrativized problem structure of WoW seeds participation structures that are inherently discursive in nature. Our constant comparative analysis indicated that Danny’s problem-solving trajectory is mediated by discursive practices oriented towards the construction and negotiation of his identity. We termed verbal descriptions used by other members within the affinity space to talk about the focal member’s discursive identities with regard to his competencies (social, gaming, academic) as discursive practices. At time 1, Danny is observed as nonchalant and unaffected by other’s construal of his player characterization. At time 2, Danny indicated self-affectedness as he recalled a conversation between his school friends who are also in WoW, who labeled him as a “looter”. A “looter” has negative connotations in that it represents a player who leverages and takes advantage of others. With respect to his relations with his peers, Danny shared that being placed in a negative light diminished his “privilege and self-worth”.

### Text 5

Danny: “…they labeled me looter, I think its unfair because I was just trying to level up as fast as I can. After they called me that, the rest did not allow me to access their cheat page, they removed my privilege….because earlier I was in there because I knew Kory. Sometimes in the canteen, I can see that Kory doesn’t want to sit with me because the rest told him that I’m making use of him which is not true…feel like so useless….no self-worth like that. They are just like that don’t know Mrs what Tang, always say bad things about me.”

The latter sentence in Text 5 indicates Danny’s resistance to be negatively regarded. In a chat session in time 3, Danny shared that within his institutionalized school context, a “certain Mrs. Tang”, his Chinese teacher, positioned him as a “slow and weak” learner. The verbal descriptions Mrs. Tang used on Danny elicited strong negative feelings, not only towards the teacher but also to the class as a whole. Through our interview with Danny’s mother, we found that although Mrs. Tang made attempts (e.g., gave him the privilege to choose his own seating arrangement in her class) to establish a rather collaborative relation with Danny, her instructional practices, which authorized only Chinese proficiency while invalidating students’ competency in other areas, failed to achieve the goal. This caused Danny to lose interest in Mrs. Tang’s Chinese class and therefore entrapping himself in a vicious cycle: acknowledges negative labeling–lose interest–does not perform–negative labeling exacerbates. We juxtaposed these insights with Danny’s performance within WoW. Within the gaming space, despite being attributed with a “looter” identity and had his privilege to certain symbolic resources removed, Danny moved on to engage in quest solutions and in establishing new synergies with other players in leveling up. If anything, the negative reception given to him by his WoW school friends enticed and incited him to perform even better than “them”.

### Text 6

Danny: “I mean they can say I’m a looter or whatever, but for example taking the mob boss down, I did not loot and my healing XP helped Runkar….SteathThrift asked me to join as their chief healer. I didn’t even post or say anything…they just asked…”

Observed here, Danny’s skills and experience in his healing class were recognized as valued symbolic resources within the game space (despite him being identified negatively by his peers). As articulated, Danny “didn’t even post or say anything…” but that did not hinder his performative enactments, characterized by non-explicit tacit knowledge, built up in repeated practice and experience (XP points), to be honored and rewarded (invitation to chief healer).

## Problem-solving and disquisitional practices

Within the contexts of affinity spaces, the participation boundaries are less structured implicating upon the massive kinds of “reifications” that may evolve as a process of participation. In conducting our constant comparative analysis, we observed that the WoW context Danny was immersed in revolved around text-walkthrough, guides, and manuals (reifications of others) that do not make sense to him unless he has experienced and lived in the game world for a while (embodied experience) to situate and construct meanings. For example, a leveling guide on the healer class did not appear lucid and meaningful to Danny until sometime after time 2, after which he has gained embodied experience through his healer characterization to situate its meaning in specific ways.

### Text 7

Danny: “…Pagin gave me his healing guide to level up to 3100DPS in 30…I did not get what he is talking about, but then after ScreeThor roped me in the boss raid, I remember Pagin doing the same boss thing, so I was reading it up for days…which really helped, we cleared 52000dps in 3secs. I just tried different ways, didn’t work, do again...”

Danny went on to create not only his own “formal” leveling guides, but also formal write-ups of his quest-solving processes. Within the gaming space, these “formal” writings, which we term as disquisitions, afford a player with not only traction as a skillful gamer but also elevates his identity to be one of the master gamers—much like being certified as a master teacher after one has fulfilled certain criteria, conditions, and certifications. At time 1, Danny’s disquisitional practice was confined to only “formal write-ups” to his guild members. At time 4, the reach of his disquisitions extended to players from other countries (such as Australians on the Oceanic server). Table 1 shows Danny’s problem-solving disquisitions over the four time lapses.

**Table 1:** Problem-solving disquisitional practices time 1 to time 4

Time 1	Time 2	Time 3	Time 4
-Healer walkthrough levels 1–10	-Healer walkthrough levels 11–15 -Healer scripting -Healer mob quest	-Paladin walkthrough 1–10 -Orc introduction -Alliance vs allies—the introduction	-Level guide 1–80 -Mob guide 1–80

In analyzing the disquisitional practices, we noted the reciprocal forms of teaching and learning that occur in all directions throughout the social network (in contrast to movement from “periphery” to “core,” Lave and Wenger 1991). This reciprocity is further underpinned by interwoven forms of competition and collaboration that appear to foster Danny’s high levels of engagement. Relating back to our observations, Danny was enthused by his peers’ negative discursiveness towards him to “compete” against them and outperform them through “collaboration” with others. Indicated in the various disquisitions at the respective time lapse, there seems to be a vital condition where although Danny has moved from periphery to the core in terms of his healer class (with its complex attributes and synergization affordances), he again moves to the periphery when it comes to appropriating the attributes of the paladin class. Against dialectics of peripheral to core and core-center to the threshold, a process of being limanised occurs. We conjecture that this process of liminalisation, of moving across thresholds, takes learners to the edge of *knowing* in that it cyclically seeds the establishment of trust, confidentiality, willingness to be within the unknown again. As Danny puts it in Text 8, it is not unlike looking at himself from “the balcony” where he can see patterns and possibly bring them into the core-center.

### Text 8

Danny: “I started of healer, then I became one of them, I was almost controlling the healer page. After that healer needs to know what the orc is damaging right. So I went into the orc space. At first I was scared to participate in case they did not welcome me, so I just quietly read their postings, but then I had to try out the DPS. Some didn’t work, I suspect they didn’t give the real truth, so I went to post. Funnily now im controlling their orc page also! But because I was reading all their posts, I know how these orcs work”

## Discussion

**Fig. 2:**
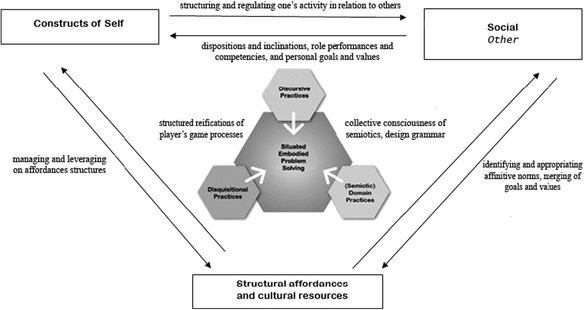
Knowing and *becoming* trajectory within the context of game learning

To this end, our investigation has shown the dialectical relations inherent between Danny (self), the game environment (structural), as well as the social context. This tripartite relationship may be further unpacked by the 3D: domain-discursive-disquisitional practices that provide an analytical lens into the problem-solving trajectory of a youth gamer in WoW, as illustrated in Fig. 2.

In classifying Danny’s game context as his affinity space, we see his shift towards making meaning of norms and disciplines and what counts as acceptable and recognizable. What is critical to note here is that, prior to time 2, as a (relatively) new gamer, Danny is of course not totally uninformed of certain norms and practices that gamers adopt. Furthermore, he has the resource of his “more advanced” gaming friends who try to tell him how to play and what to do, but Danny was seemingly uninterested and never valued their “instructions”/“directions”/“guidance” (see Text 2). However, over time, as he persists in engaging in the tripartite problem-solving structure of quest-instance-raid, the designed experiences affords a trajectory where he is able to evaluate the results of his own “probes” within the domain through practice and interaction with the affinity group. This probing-enacting cycle seeds Danny’s appreciative system for the affinity domain—this is to say through practice and interaction, Danny comes to recognize and reflect overtly on the goals and desires that characterize the domain. He does not only appropriate skills and knowledge that are scientifically and mathematically computational in nature but also begins to appreciate the identity, epistemology, and values, the grammar as Gee (2007) terms it, of the domain space. His recognition and reflection of the space is evidenced by disquisitional artifacts of metalevel analysis and reflection of the gameplay procedures. Not only does he provide solutions to the respective questing and raiding problems, he also critique games and imagine new and different add-ons within the disquisitions. 

Several implications from this study may be drawn for institutionalized learning within the STEM domain. Specifically, the findings indicate how meaning-making in the narrativized structures of WoW is characterized by processes of narrativity that underpin the three nested levels as domain-related, discursive, and disquisitional practices, as captured in Fig. 2.

Within the context of WoW as a relational game play, domain practices, in Gee’s (2003) semiotic domain sense, were defined as those which bear upon the construction of domain identities, such as learned norms and the merging of goals and values that merged with the player’s own. This may include enactments out of the gaming space but within related WoW sites such as the WoW forum page. Disquisitional practices referred to more structured reifications of player’s game play processes, such as the creation of “leveling up” guides for game players to progress to more advanced levels of play and co-creation of game play raid groups to achieve a common goal within the game. Discursive practices referred to verbal descriptions used by both the player and his social others with regard to a player’s identity in terms of his performance and competencies. For instance, this may be reflected in player’s articulation of the recognition of another player as an “expert”, based on his or her skillful gameplay sets. Within this vein, textual and verbal enactments by both the player and his social others with regard to a player’s identity in terms of his performance and competencies were coded as discursive practices. At the basal level, we posit that learning and *knowing* in WoW is inherently and inextricably linked with concept of *self* and the emergence of conscious control over one’s actions. This includes cognizance of one’s abilities in enacting performance within a particular context and a consciousness in terms of one’s actions and motives to confront new challenges and goals and willingness to learn, unlearn, and relearn (reflective and regulatory capabilities). When individuals are faced with new challenges, meanings in the form of reifications are constructed as to what these goals mean in terms of performance and action. These reifications become psychological artifacts from which cognition and action are mediated. The appropriate development of one’s concept of self is thus critical towards greater expertise in one’s metacognitive and self-reflective capabilities. Mead (1934) explicated the concept of self as a social construct that involves a level of consciousness of being regarded as self by social others. Moving away from theories of objectivity, there is therefore the situated notion of subjectivity in terms of one’s metacognitive knowledge as arising from the concept of self. Recourse to social constructivist accounts of cognitive development allows us to understand the interplay between game players’ embodied knowing trajectories and constructs of *self* as observed within the WoW space. Social constructivism posits that knowledge is constructed from social interactions and meaning-making processes. In other words, whereas knowledge is socially constructed as a meaning-making process, identity is the social construction of meaning-making about one’s *self*. At a quotidian level, one’s development of a higher order of cognitive awareness in life (metacognitive) thus may ultimately hinge on prior social consciousness or what Ricouer (1992) terms as identifying “one self as another”.

Building on this notion of higher order cognitive structures, as arising from the interplay between self and social others, links to broader notions of regulatory and performative behavior (Vygotsky 1978). While social entailments to learning and knowledge creation have been heavily foregrounded in constructivist theories, it still remains inadequately explicated how aspects of sociality impinge upon a sense of socially mediated metacognition. As observed from the ethnographic observation, Danny was constantly involved within the flux of socially mediated webs. At the crux of notions of social mediation lies the construction of intersubjective spaces that stems not only a sense of self but also sets of coordinated interrelations that seed leveling up cognizance, affordance management, and his computational understandings of the workings of the game. Danny’s learning occurs within collective phenomena of social identities, social knowledge, and social movement—a collective consciousness of the (semiotic) *domain* of interaction. Yet, paradoxically, much of the development of work in social constructivist theories has been rooted at the individual level of analysis—even collective phenomena regarded as evolved in social spaces were understood in terms of individual attitudes, emotions, thinking, reasoning, and cognitive processes with social factors recognized as “mere variables” (Jovchelovitch 2006, p.72). Recognizing that knowledge formation is itself at the core of sociality, we posit that the emergence of higher order cognitive structures such as metacognition in knowing trajectories thus, too, rely on the interplay between self and the social.

Additionally, Danny was observed to lean heavily on the WoW design and environmental structure for cognitive support. This seeds the construal of a third self-socio-structural nested level, other than self and social, within our characterizations. 

While Danny started off playing WoW from a simple gameplay of attempting to survive till the end-game level, his *knowing and meaning-making* trajectory entails a developmental cognizance about (i) how his character has fared, (ii) the discursive identities surrounding his character, (iii) simplifying the gameplay process for *others*, and (iv) caring enough to impact upon the game design through his respective disquisitions. In a sense through the triadic interplay between self-social and the structural, underpinned by domain-disquisitions-discursive practices, Danny was able to form his own “meta trajectory” appropriated through enactive embodying practices of game play, as arising from technology-afforded problem-solving interplays. Importantly, we posit that Danny’s trajectory represents a microcosm of the crux of *knowing* and *becoming* that are integral for the fundamental basis of problem-solving for STEM learning.
